# Supplementary material for: MitoQ as an antenatal antioxidant treatment improves markers of lung maturation in healthy and hypoxic pregnancy
Source: J Physiol. 2023 Jul 19;601(16):3647–65. doi: 10.1113/JP284786 (PMC10952154; doi:10.1113/JP284786)
Supplement: Supplementary file 1 — Statistical Summary Document [file TJP-601-3647-s001.docx]

**Manuscript Title:** MitoQ as an antenatal antioxidant treatment improves lung development in healthy and hypoxic pregnancy

**Authors:** Mitchell C. Lock, Kimberley J. Botting, Beth J. Allison, Youguo Niu, Sage G. Ford, Michael P. Murphy, Sandra Orgeig, Dino A. Giussani*, Janna L. Morrison*

**Animal model used, if applicable:** Welsh mountain Sheep

**Underlying hypothesis:** Here, we tested the hypothesis that maternal treatment with MitoQ in late gestation will improve maturation of the developing lung in both normal and hypoxic pregnancy in sheep, a species with similar fetal lung developmental milestones as humans

**Definitions of ‘n’:**

n = number of animals per specified treatment group. A total of 36 Welsh mountain ewes carrying singleton pregnancies were used in this study.

**Statistical summary table:**

| Experimental question number* | Finding/ conclusion | Experimental location/ variable  e.g. cortex vs cerebellum or genotype | Mean value  (or other summary statistic) | SD | n (value) | P**  H = Hypoxia  M = MitoQ  Int = Interaction | Units | Data comparisons  e.g. WT vs KO | Statistical test | Figure/table in which data are presented |
| --- | --- | --- | --- | --- | --- | --- | --- | --- | --- | --- |
| 1. Fetal Body weight | There was a reduction in fetal body weight in hypoxic fetuses | Whole Fetal Weight | NS: 3.931 NM: 4.21HS: 3.319 HM: 3.33 | NS: 0.3976 NM: 0.7117 HS: 0.9335 HM: 0.6192 | NS: 7 NM: 8 HS: 10 HM: 10 | H: **0.0033** M: 0.7134 Int: 0.461 | Kg | Normoxia vs Hypoxia & Saline vs MitoQ | 2-way ANOVA | Figure 1 |
| 2.  Lung Weight | There was a reduction in lung weight in hypoxic fetuses | Lungs | NS: 106.8 NM: 99.76 HS: 75.67 HM: 76.66 | NS: 20.18 NM: 6.941 HS: 22 HM: 16.58 | NS: 7 NM: 8 HS: 10 HM: 10 | H: **0.0001** M: 0.5999 Int: 0.4874 | g | Normoxia vs Hypoxia & Saline vs MitoQ | 2-way ANOVA | Figure 1 |
| 3. Relative lung weight | did not change between groups | Lungs | NS: 2.712 NM: 2.425 HS: 2.293 HM: 2.385 | NS: 0.4022 NM: 0.4097 HS: 0.2687 HM: 0.3473 | NS: 7 NM: 8 HS: 10 HM: 10 | H: 0.7164 M: 0.1297 Int: 0.9516 | g/Kg | Normoxia vs Hypoxia & Saline vs MitoQ | 2-way ANOVA | Figure 1 |
| 4. SFTPA mRNA | did not change between groups | Lungs | NS: 0.591 NM: 0.567 HS: 0.599 HM: 0.622 | NS: 0.276 NM: 0.218 HS: 0.108 HM: 0.223 | NS: 7 NM: 7 HS: 10 HM: 10 | H: 0.6786 M: 0.9983 Int: 0.7597 | MNE | Normoxia vs Hypoxia & Saline vs MitoQ | 2-way ANOVA | Table 2 |
| 5. SFTPB mRNA | There was an increase in SPB mRNA expression caused by hypoxia and MitoQ | Lungs | NS: 1.504 NM: 1.753 HS: 1.893 HM: 2.075 | NS: 0.343 NM: 0.371 HS: 0.245 HM: 0.581 | NS: 6 NM: 7 HS: 9 HM: 10 | H: **0.011** M: **0.0291** Int: 0.8879 | MNE | Normoxia vs Hypoxia & Saline vs MitoQ | 2-way ANOVA | Table 2 |
| 6. SFTPC mRNA | There was an increase in SPC mRNA expression caused by MitoQ | Lungs | NS: 4.164 NM: 6.142 HS: 5.254 HM: 6.032 | NS: 1.147 NM: 1.664 HS: 0.807 HM: 2.272 | NS: 6 NM: 7 HS: 10 HM: 10 | H: 0.4206 **M: 0.029** Int: 0.325 | MNE | Normoxia vs Hypoxia & Saline vs MitoQ | 2-way ANOVA | Table 2 |
| 7. SFTPD mRNA | There was an increase in SP-D mRNA expression in the hypoxia-MitoQ group | Lungs | NS: 0.030 NM: 0.031 HS: 0.031 HM: 0.044 | NS: 0.014 NM: 0.011 HS: 0.011 HM: 0.015 | NS: 7 NM: 8 HS: 10 HM: 9 | H: 0.089 M: 0.1147 Int: **0.0170** | MNE | Normoxia vs Hypoxia & Saline vs MitoQ | 2-way ANOVA | Table 2 |
| 8. PCYT1A mRNA | There was an increase in PCYT1A caused by Hypoxia and a decrease caused by MitoQ | Lungs | NS: 0.043 NM: 0.039 HS: 0.049 HM: 0.042 | NS: 0.005 NM: 0.004 HS: 0.011 HM: 0.006 | NS: 7 NM: 8 HS: 9 HM: 10 | H: **0.0271** M: **0.0249** Int: 0.4881 | MNE | Normoxia vs Hypoxia & Saline vs MitoQ | 2-way ANOVA | Table 2 |
| 9. ABCA3 mRNA | did not change between groups | Lungs | NS: 0.093 NM: 0.102 HS: 0.101 HM: 0.116 | NS: 0.032 NM: 0.025 HS: 0.028 HM: 0.030 | NS: 7 NM: 8 HS: 10 HM: 10 | H: 0.2708 M: 0.2051 Int: 0.7428 | MNE | Normoxia vs Hypoxia & Saline vs MitoQ | 2-way ANOVA | Table 2 |
| 10. SP-B (AU) | There was an increase in SPB protein expression caused by hypoxia and MitoQ | Lungs | NS: 0.0220 NM: 0.0263 HS: 0.0339 HM: 0.0704 | NS: 0.0108 NM: 0.0146 HS: 0.0320 HM: 0.0333 | NS: 7 NM: 7 HS: 6 HM: 6 | H: **0.0071** M: **0.0417** Int: 0.1023 | AU | Normoxia vs Hypoxia & Saline vs MitoQ | 2-way ANOVA | Table 2 |
| 11. TTF1 mRNA | There was a decrease in TTF-1 mRNA in the hypoxia-saline group | Lungs | NS: 0.151 NM: 0.115 HS: 0.091 HM: 0.149 | NS: 0.059 NM: 0.032 HS: 0.013 HM: 0.038 | NS: 6 NM: 6 HS: 10 HM: 10 | H: 0.3528 M: 0.4637 Int: **0.0021** | MNE | Normoxia vs Hypoxia & Saline vs MitoQ | 2-way ANOVA | Table 2 |
| 12. FOXA1 mRNA | did not change between groups | Lungs | NS: 0.001 NM: 0.001 HS: 0.001 HM: 0.001 | NS: 0.0003 NM: 0.0003 HS: 0.0002 HM: 0.0003 | NS: 7 NM: 8 HS: 10 HM: 10 | H: 0.2761 M: 0.9364 Int: 0.3199 | MNE | Normoxia vs Hypoxia & Saline vs MitoQ | 2-way ANOVA | Table 2 |
| 13. SP1 mRNA | did not change between groups | Lungs | NS: 0.281 NM: 0.236 HS: 0.262 HM: 0.261 | NS: 0.032 NM: 0.040 HS: 0.034 HM: 0.039 | NS: 7 NM: 8 HS: 10 HM: 10 | H: 0.8343 M: 0.0779 Int: 0.0951 | MNE | Normoxia vs Hypoxia & Saline vs MitoQ | 2-way ANOVA | Table 2 |
| 14. TTF-1 (AU) | Hypoxia increased protein expression of TTF-1 | Lungs | NS: 0.123 NM: 0.130 HS: 0.217 HM: 0.175 | NS: 0.0607 NM: 0.0580 HS: 0.0967 HM: 0.0602 | NS: 7 NM: 7 HS: 6 HM: 7 | H: **0.0153** M: 0.5425 Int: 0.1753 | AU | Normoxia vs Hypoxia & Saline vs MitoQ | 2-way ANOVA | Table 2 |
| 15. NR3C1 mRNA | did not change between groups | Lungs | NS: 0.200 NM: 0.176 HS: 0.184 HM: 0.198 | NS: 0.031 NM: 0.031 HS: 0.027 HM: 0.025 | NS: 7 NM: 8 HS: 10 HM: 10 | H: 0.7433 M: 0.6208 Int: 0.0578 | MNE | Normoxia vs Hypoxia & Saline vs MitoQ | 2-way ANOVA | Table 2 |
| 16. NR3C2 mRNA | did not change between groups | Lungs | NS: 0.008 NM: 0.006 HS: 0.007 HM: 0.007 | NS: 0.002 NM: 0.001 HS: 0.002 HM: 0.002 | NS: 7 NM: 8 HS: 9 HM: 10 | H: 0.6041 M: 0.4561 Int: 0.3493 | MNE | Normoxia vs Hypoxia & Saline vs MitoQ | 2-way ANOVA | Table 2 |
| 17. HSD11B1 mRNA | did not change between groups | Lungs | NS: 0.013 NM: 0.012 HS: 0.013 HM: 0.014 | NS: 0.005 NM: 0.005 HS: 0.003 HM: 0.004 | NS: 7 NM: 8 HS: 10 HM: 10 | H: 0.4456 M: 0.9681 Int: 0.5636 | MNE | Normoxia vs Hypoxia & Saline vs MitoQ | 2-way ANOVA | Table 2 |
| 18. HSD11B2 mRNA | There was a decrease in HSD11B2 mRNA expression caused by Hypoxia and an increase caused by MitoQ | Lungs | NS: 0.003 NM: 0.004 HS: 0.002 HM: 0.003 | NS: 0.001 NM: 0.001 HS: 0.001 HM: 0.0004 | NS: 7 NM: 7 HS: 10 HM: 10 | H: **0.0022** M: **0.0498** Int: 0.5106 | MNE | Normoxia vs Hypoxia & Saline vs MitoQ | 2-way ANOVA | Table 2 |
| 19. HIF3A mRNA | There was an increase in HIF3A mRNA expression caused by hypoxia | Lungs | NS: 0.031 NM: 0.030 HS: 0.047 HM: 0.056 | NS: 0.008 NM: 0.007 HS: 0.013 HM: 0.020 | NS: 6 NM: 8 HS: 8 HM: 10 | H: **0.0002** M: 0.421 Int: 0.3416 | MNE | Normoxia vs Hypoxia & Saline vs MitoQ | 2-way ANOVA | Table 2 |
| 20. EGLN3 mRNA | There was an increase in EGLN3 mRNA expression caused by hypoxia | Lungs | NS: 0.008 NM: 0.012 HS: 0.020 HM: 0.024 | NS: 0.003 NM: 0.006 HS: 0.009 HM: 0.010 | NS: 6 NM: 6 HS: 10 HM: 10 | H: 0.0003 M: 0.121 Int: 0.9642 | MNE | Normoxia vs Hypoxia & Saline vs MitoQ | 2-way ANOVA | Table 2 |
| 21. EGLN1 (AU) | did not change between groups | Lungs | NS: 0.0070 NM: 0.0063 HS: 0.0065 HM: 0.0046 | NS: 0.0062 NM: 0.0023 HS: 0.0063 HM: 0.0042 | NS: 7 NM: 7 HS: 6 HM: 7 | H: 0.7822 M: 0.6994 Int: 0.9805 | AU | Normoxia vs Hypoxia & Saline vs MitoQ | 2-way ANOVA | Table 2 |
| 22. EGLN3 (AU) | did not change between groups | Lungs | NS: 0.0547 NM: 0.0506 HS: 0.055 HM: 0.0517 | NS: 0.0055 NM: 0.0070 HS: 0.0084 HM: 0.0050 | NS: 7 NM: 7 HS: 6 HM: 7 | H: 0.7164 M: 0.1297 Int: 0.9516 | AU | Normoxia vs Hypoxia & Saline vs MitoQ | 2-way ANOVA | Table 2 |
| 23. INOS mRNA | INOS mRNA was downregulated in hypoxia-saline but upregulated hypoxia-mitoQ | Lungs | NS: 0.053 NM: 0.048 HS: 0.041 HM: 0.057 | NS: 0.011 NM: 0.010 HS: 0.005 HM: 0.010 | NS: 6 NM: 7 HS: 10 HM: 9 | H: 0.3912 M: 0.4095 Int: 0.0495 | MNE | Normoxia vs Hypoxia & Saline vs MitoQ | 2-way ANOVA | Table 2 |
| 24. ENOS mRNA | There was a decrease in ENOS mRNA expression caused by MitoQ | Lungs | NS: 0.017 NM: 0.014 HS: 0.016 HM: 0.014 | NS: 0.002 NM: 0.003 HS: 0.004 HM: 0.003 | NS: 7 NM: 8 HS: 9 HM: 10 | H: 0.4957 M: **0.0121** Int: 0.4829 | MNE | Normoxia vs Hypoxia & Saline vs MitoQ | 2-way ANOVA | Table 2 |
| 25. TFAM mRNA | did not change between groups | Lungs | NS: 0.067 NM: 0.061 HS: 0.067 HM: 0.072 | NS: 0.011 NM: 0.010 HS: 0.008 HM: 0.006 | NS: 7 NM: 8 HS: 10 HM: 10 | H: 0.4254 M: 0.0615 Int: 0.0132 | MNE | Normoxia vs Hypoxia & Saline vs MitoQ | 2-way ANOVA | Table 2 |
| 26. NRF1 mRNA | There was an increase in NRF1 mRNA epression caused by hypoxia | Lungs | NS: 0.036 NM: 0.031 HS: 0.039 HM: 0.041 | NS: 0.002 NM: 0.006 HS: 0.006 HM: 0.005 | NS: 6 NM: 8 HS: 10 HM: 9 | H: **0.0026** M: 0.3052 Int: 0.1061 | MNE | Normoxia vs Hypoxia & Saline vs MitoQ | 2-way ANOVA | Table 2 |
| 27. Complex I (AU) | There was a reduction in Complex I expression caused by Hypoxia | Lungs | NS: 0.0078 NM: 0.0090 HS: 0.0054 HM: 0.0057 | NS: 0.0037 NM: 0.0017 HS: 0.0015 HM: 0.0022 | NS: 7 NM: 7 HS: 6 HM: 6 | H: **0.0074** M: 0.4461 Int: 0.6385 | AU | Normoxia vs Hypoxia & Saline vs MitoQ | 2-way ANOVA | Table 2 |
| 28. Complex II (AU) | did not change between groups | Lungs | NS: 0.0023 NM: 0.0028 HS: 0.0022 HM: 0.0023 | NS: 0.0006 NM: 0.0003 HS: 0.0003 HM: 0.0008 | NS: 7 NM: 7 HS: 6 HM: 7 | H: 0.1908 M: 0.1338 Int: 0.5472 | AU | Normoxia vs Hypoxia & Saline vs MitoQ | 2-way ANOVA | Table 2 |
| 29. Complex III (AU) | There was an increase in Complex III expression caused by MitoQ | Lungs | NS: 0.0057 NM: 0.0076 HS: 0.0054 HM: 0.0063 | NS: 0.0017 NM: 0.0009 HS: 0.0008 HM: 0.0020 | NS: 7 NM: 7 HS: 6 HM: 7 | H: 0.1655 M: **0.0271** Int: 0.398 | AU | Normoxia vs Hypoxia & Saline vs MitoQ | 2-way ANOVA | Table 2 |
| 30. Complex IV (AU) | did not change between groups | Lungs | NS: 0.0004 NM: 0.0005 HS: 0.0003 HM: 0.0004 | NS: 0.0001 NM: 0.0002 HS: 6e-005 HM: 0.0002 | NS: 7 NM: 7 HS: 6 HM: 7 | H: 0.0855 M: 0.2949 Int: 0.5726 | AU | Normoxia vs Hypoxia & Saline vs MitoQ | 2-way ANOVA | Table 2 |
| 31. Complex V (AU) | There was an increase in Complex V expression caused by MitoQ | Lungs | NS: 0.0063 NM: 0.0075 HS: 0.0056 HM: 0.0067 | NS: 0.0019 NM: 0.0013 HS: 0.0010 HM: 0.0023 | NS: 7 NM: 7 HS: 6 HM: 7 | H: 0.2547 M: **0.0482** Int: 0.8942 | AU | Normoxia vs Hypoxia & Saline vs MitoQ | 2-way ANOVA | Table 2 |
| 32. Mitobiogenesis | did not change between groups | Lungs | NS: 0.1300 NM: 0.1606 HS: 0.1361 HM: 0.1449 | NS: 0.0458 NM: 0.0563 HS: 0.0486 HM: 0.0834 | NS: 7 NM: 7 HS: 6 HM: 7 | H: 0.8393 M: 0.4096 Int: 0.6461 | AU | Normoxia vs Hypoxia & Saline vs MitoQ | 2-way ANOVA | Table 2 |
| 33. HMOX1 mRNA | There was a reduction in HMOX1 mRNA caused by MitoQ | Lungs | NS: 0.037 NM: 0.032 HS: 0.039 HM: 0.029 | NS: 0.012 NM: 0.009 HS: 0.010 HM: 0.008 | NS: 7 NM: 8 HS: 9 HM: 9 | H: 0.8961 M: **0.048** Int: 0.4241 | MNE | Normoxia vs Hypoxia & Saline vs MitoQ | 2-way ANOVA | Table 2 |
| 34. NOX4 mRNA | There was a reduction in NOX4 mRNA expression caused by Hypoxia | Lungs | NS: 0.004 NM: 0.005 HS: 0.003 HM: 0.003 | NS: 0.002 NM: 0.002 HS: 0.001 HM: 0.001 | NS: 7 NM: 8 HS: 10 HM: 10 | H: **0.0081** M: 0.4408 Int: 0.5246 | MNE | Normoxia vs Hypoxia & Saline vs MitoQ | 2-way ANOVA | Table 2 |
| 35. CAT mRNA | did not change between groups | Lungs | NS: 0.151 NM: 0.150 HS: 0.158 HM: 0.145 | NS: 0.025 NM: 0.027 HS: 0.017 HM: 0.029 | NS: 7 NM: 7 HS: 10 HM: 10 | H: 0.8951 M: 0.4464 Int: 0.5295 | MNE | Normoxia vs Hypoxia & Saline vs MitoQ | 2-way ANOVA | Table 2 |
| 36. GPX mRNA | did not change between groups | Lungs | NS: 0.013 NM: 0.010 HS: 0.016 HM: 0.013 | NS: 0.005 NM: 0.003 HS: 0.007 HM: 0.003 | NS: 7 NM: 8 HS: 9 HM: 9 | H: 0.0986 M: 0.0897 Int: 0.7261 | MNE | Normoxia vs Hypoxia & Saline vs MitoQ | 2-way ANOVA | Table 2 |
| 37. SOD1 mRNA | did not change between groups | Lungs | NS: 0.337 NM: 0.330 HS: 0.348 HM: 0.316 | NS: 0.070 NM: 0.073 HS: 0.009 HM: 0.027 | NS: 7 NM: 6 HS: 9 HM: 10 | H: 0.9319 M: 0.3118 Int: 0.5177 | MNE | Normoxia vs Hypoxia & Saline vs MitoQ | 2-way ANOVA | Table 2 |
| 38. SOD2 mRNA | There was an increase in SOD2 mRNA caused by hypoxia | Lungs | NS: 0.064 NM: 0.064 HS: 0.077 HM: 0.088 | NS: 0.018 NM: 0.014 HS: 0.013 HM: 0.015 | NS: 7 NM: 8 HS: 10 HM: 10 | H: **0.0006** M: 0.5257 Int: 0.1564 | MNE | Normoxia vs Hypoxia & Saline vs MitoQ | 2-way ANOVA | Table 2 |
| 39. SOD (AU) | did not change between groups | Lungs | NS: 0.0114 NM: 0.0122 HS: 0.0092 HM: 0.0106 | NS: 0.0033 NM: 0.0035 HS: 0.0026 HM: 0.0021 | NS: 7 NM: 7 HS: 6 HM: 7 | H: 0.3217 M: 0.1092 Int: 0.8114 | AU | Normoxia vs Hypoxia & Saline vs MitoQ | 2-way ANOVA | Table 2 |
| 40. AQP1 mRNA | There was an increase in AQP1 mRNA caused by hypoxia | Lungs | NS: 0.126 NM: 0.146 HS: 0.190 HM: 0.165 | NS: 0.020 NM: 0.029 HS: 0.049 HM: 0.037 | NS: 6 NM: 7 HS: 9 HM: 10 | H: **0.0032** M: 0.8661 Int: 0.0497 | MNE | Normoxia vs Hypoxia & Saline vs MitoQ | 2-way ANOVA | Table 2 |
| 41. AQP2 mRNA | There was an increase in AQP2 mRNA caused by hypoxia | Lungs | NS: 0.0004 NM: 0.0004 HS: 0.0006 HM: 0.0006 | NS: 0.00003 NM: 0.0001 HS: 0.0002 HM: 0.0002 | NS: 5 NM: 7 HS: 10 HM: 10 | H: **0.0043** M: 0.3744 Int: 0.6583 | MNE | Normoxia vs Hypoxia & Saline vs MitoQ | 2-way ANOVA | Table 2 |
| 42. AQP4 mRNA | did not change between groups | Lungs | NS: 0.010 NM: 0.013 HS: 0.015 HM: 0.014 | NS: 0.001 NM: 0.008 HS: 0.009 HM: 0.008 | NS: 5 NM: 8 HS: 9 HM: 10 | H: 0.3357 M: 0.646 Int: 0.4681 | MNE | Normoxia vs Hypoxia & Saline vs MitoQ | 2-way ANOVA | Table 2 |
| 43. ATP1A1 mRNA | ATP1A1 mRNA was increased in the hypoxia-saline vs normoxia-saline | Lungs | NS: 0.051 NM: 0.058 HS: 0.055 HM: 0.058 | NS: 0.015 NM: 0.008 HS: 0.015 HM: 0.011 | NS: 5 NM: 8 HS: 9 HM: 10 | H: 0.2521 M: 0.0567 Int: **0.0435** | MNE | Normoxia vs Hypoxia & Saline vs MitoQ | 2-way ANOVA | Table 2 |
| 44. SCNN1A mRNA | SCNN1A mRNA was increased in the hypoxia-mitoQ vs normoxia-mitoQ | Lungs | NS: 0.015 NM: 0.013 HS: 0.018 HM: 0.021 | NS: 0.005 NM: 0.004 HS: 0.005 HM: 0.007 | NS: 7 NM: 7 HS: 9 HM: 9 | H: 0.2529 M: 0.6906 Int: **0.0313** | MNE | Normoxia vs Hypoxia & Saline vs MitoQ | 2-way ANOVA | Table 2 |
| 45. ATP1A1 (AU) | ATP1A1 protein expression was reduced by mitoQ | Lungs | NS: 0.0798 NM: 0.0653 HS: 0.0869 HM: 0.0382 | NS: 0.0317 NM: 0.0256 HS: 0.0355 HM: 0.0114 | NS: 7 NM: 7 HS: 6 HM: 7 | H: 0.3506 M: **0.0062** Int: 0.1166 | AU | Normoxia vs Hypoxia & Saline vs MitoQ | 2-way ANOVA | Table 2 |
| 46. ERN1 mRNA | did not change between groups | Lungs | NS: 0.014 NM: 0.015 HS: 0.014 HM: 0.016 | NS: 0.002 NM: 0.003 HS: 0.002 HM: 0.005 | NS: 6 NM: 8 HS: 10 HM: 10 | H: 0.2824 M: 0.4214 Int: 0.5545 | MNE | Normoxia vs Hypoxia & Saline vs MitoQ | 2-way ANOVA | Table 2 |
| 47. ATF6 mRNA | did not change between groups | Lungs | NS: 0.149 NM: 0.128 HS: 0.104 HM: 0.132 | NS: 0.041 NM: 0.043 HS: 0.008 HM: 0.043 | NS: 6 NM: 6 HS: 8 HM: 10 | H: 0.16 M: 0.8137 Int: 0.0975 | MNE | Normoxia vs Hypoxia & Saline vs MitoQ | 2-way ANOVA | Table 2 |
| 48. EIF2AK3 mRNA | did not change between groups | Lungs | NS: 0.035 NM: 0.028 HS: 0.033 HM: 0.034 | NS: 0.007 NM: 0.007 HS: 0.004 HM: 0.003 | NS: 7 NM: 8 HS: 10 HM: 10 | H: 0.2548 M: 0.1044 Int: **0.0483** | MNE | Normoxia vs Hypoxia & Saline vs MitoQ | 2-way ANOVA | Table 2 |
| 49. Cortisol (ng/ml/mg) | did not change between groups | Lungs | NS: 0.0016 NM: 0.0021 HS: 0.0023 HM: 0.0027 | NS: 0.0010 NM: 0.0006 HS: 0.0013 HM: 0.0013 | NS: 7 NM: 6 HS: 6 HM: 7 | H: 0.1369 M: 0.3076 Int: 0.9726 | ng/ml/mg | Normoxia vs Hypoxia & Saline vs MitoQ | 2-way ANOVA | Table 3 |
| 50. Cortisone (ng/ml/mg) | did not change between groups | Lungs | NS: 0.0036 NM: 0.0027 HS: 0.0031 HM: 0.0029 | NS: 0.0009 NM: 0.0010 HS: 0.0009 HM: 0.0007 | NS: 6 NM: 5 HS: 6 HM: 7 | H: 0.7025 M: 0.1848 Int: 0.3407 | ng/ml/mg | Normoxia vs Hypoxia & Saline vs MitoQ | 2-way ANOVA | Table 3 |
| 51. Progesterone (ng/ml/mg) | Progesterone tissue concentration was reduced by MitoQ treatment | Lungs | NS: 0.0046 NM: 0.0026 HS: 0.0035 HM: 0.0023 | NS: 0.0018 NM: 0.0001 HS: 0.0021 HM: 0.0007 | NS: 7 NM: 6 HS: 6 HM: 7 | H: 0.2327 M: **0.0094** Int: 0.562 | ng/ml/mg | Normoxia vs Hypoxia & Saline vs MitoQ | 2-way ANOVA | Table 3 |
| 52. T3 (ng/ml/mg) | T3 tissue concentration was reduced by Hypoxia | Lungs | NS: 0.0004 NM: 0.0004 HS: 0.0004 HM: 0.0003 | NS: 6e-005 NM: 4e-005 HS: 4e-005 HM: 6e-005 | NS: 6 NM: 6 HS: 6 HM: 7 | H: **0.032** M: 0.2756 Int: 0.9392 | ng/ml/mg | Normoxia vs Hypoxia & Saline vs MitoQ | 2-way ANOVA | Table 3 |
| 53. T4 (ng/ml/mg) | T4 tissue concentration was reduced by Hypoxia | Lungs | NS: 0.0247 NM: 0.0142 HS: 0.0098 HM: 0.0124 | NS: 0.0143 NM: 0.0029 HS: 0.0028 HM: 0.0059 | NS: 7 NM: 6 HS: 6 HM: 7 | H: **0.0189** M: 0.2386 Int: 0.0575 | ng/ml/mg | Normoxia vs Hypoxia & Saline vs MitoQ | 2-way ANOVA | Table 3 |

*You may use multiple lines for the same question to indicate multiple comparisons

** Authors may wish to make the text bold where p is considered significant against a stated confidence limit.
